# Changes in the Diversity of Soil Arbuscular Mycorrhizal Fungi after Cultivation for Biofuel Production in a Guantanamo (Cuba) Tropical System

**DOI:** 10.1371/journal.pone.0034887

**Published:** 2012-04-19

**Authors:** Maria del Mar Alguacil, Emma Torrecillas, Guillermina Hernández, Antonio Roldán

**Affiliations:** 1 CSIC-Centro de Edafología y Biología Aplicada del Segura, Department of Soil and Water Conservation, Campus de Espinardo, Murcia, Spain; 2 Instituto de Ecología y Sistemática-CITMA, Grupo de Ecología del Suelo, Capdevila Rancho Boyeros, La Habana, Cuba; University of Tartu, Estonia

## Abstract

The arbuscular mycorrhizal fungi (AMF) are a key, integral component of the stability, sustainability and functioning of ecosystems. In this study, we characterised the AMF biodiversity in a native vegetation soil and in a soil cultivated with *Jatropha curcas* or *Ricinus communis*, in a tropical system in Guantanamo (Cuba), in order to verify if a change of land use to biofuel plant production had any effect on the AMF communities. We also asses whether some soil properties related with the soil fertility (total N, Organic C, microbial biomass C, aggregate stability percentage, pH and electrical conductivity) were changed with the cultivation of both crop species. The AM fungal small sub-unit (SSU) rRNA genes were subjected to PCR, cloning, sequencing and phylogenetic analyses. Twenty AM fungal sequence types were identified: 19 belong to the Glomeraceae and one to the Paraglomeraceae. Two AMF sequence types related to cultured AMF species (Glo G3 for *Glomus sinuosum* and Glo G6 for *Glomus intraradices*-*G. fasciculatum-G. irregulare*) did not occur in the soil cultivated with *J. curcas* and *R. communis*. The soil properties (total N, Organic C and microbial biomass C) were higher in the soil cultivated with the two plant species. The diversity of the AMF community decreased in the soil of both crops, with respect to the native vegetation soil, and varied significantly depending on the crop species planted. Thus, *R. communis* soil showed higher AMF diversity than *J. curcas* soil. In conclusion, *R. communis* could be more suitable for the long-term conservation and sustainable management of these tropical ecosytems.

## Introduction

The exhaustion of oil reserves and requirements for the protection of the environment, together with increasing diesel fuel prices, have made it necessary to find new sources of energy. An alternative that is currently booming worldwide is fuel obtained from renewable resources or “biofuel”. In several countries around the world, plant species such as *Jatropha curcas* and *Ricinus communis* are being used to produce biodiesel [1], [2], [3], [4]. These species, native to Africa and Central America, respectively, both belong to the Euphorbiaceae family and are widely distributed in wild or semi-cultivated areas in tropical and sub-tropical countries such as Central and South America, Africa, India and South East Asia [5]. They are small trees/shrubs, well-adapted to arid conditions and able to withstand long periods of drought. Both species are multi-purpose, since besides biodiesel production they are also used for traditional medicine, hedging fences and preventing soil erosion.

Productive plant communities require a supportive soil microbial community; this is especially important for sustainable, low-cost biofuel production on marginal lands. Within the soil microbial community, the arbuscular mycorrhizal fungi (AMF) are the most-important symbionts, being a key, integral component of plant communities in both natural and agricultural ecosystems. The AMF form symbiotic relationships with the majority of land plants, including many crops [6]. They play a vital role in plant growth and productivity since they provide nutrients, especially phosphorus, to plants and, in return, receive plant carbon assimilates [6]. Moreover, AMF protect their hosts from pathogens [7], [8] and drought [9]. Also, it has been shown that AMF improve the soil structure [10], [11], thus helping to enhance the sustainability of ecosystems. Moreover, plant diversity and productivity in ecosystems are influenced significantly by the AM fungal diversity in the soil [12]. Therefore, knowledge of the natural diversity of AMF in the root-associated soil of established biofuel-crop plantations is essential for better management, sustainability and productivity of these tropical ecosystems.

Until now very few studies have been carried out with AMF and these two crop species. It has been reported that *J. curcas* crops are highly colonised by AMF in northern Thailand [13] and that inoculation of *J. curcas* seedlings with AMF can promote their establishment under NaCl-induced stress [14]. In other study a positive response to mycorrhizal inoculation in the rhizosphere of *J. curcas* has been reported [15]. However, in spite of their relevance to the sustainability of the ecosystems in a global change basis, the effects of biofuel plant production on the populations and diversity of AMF remain unknown. Therefore, the objective of this study was to assess, through a molecular approach, the changes in the biodiversity of AMF mediated by the cultivation of two biofuel crops, *J. curcas* and *R. communis*, in a tropical system.

## Results

### Soil properties

The pH and electrical conductivity did not differ significantly between the native vegetation soil and soils planted with *J. curcas* or *R. communis* (Table 1). The soil total N, total organic C and concentration of microbial biomass C were significantly higher in the soil planted with both crop species with respect to native vegetation soil; however, the aggregate stability was significantly lower.

**Table 1:** Biological, chemical and physical properties of native vegetation soil and *Ricinus communis* and *Jatropa curcas* soil (n = 5).

	Native vegetation soil	*R. communis* soil	*J. curcas* soil
Total N (g Kg^−1^)	3.67±0.01b	5.67±0.03a	4.59±0.02a
Organic C (%)	1.76±0.02c	2.48±0.01a	2.11±0.01b
Aggregate stability (%)	64.1±0.42a	46.3±3.64b	53.1±1.55b
MBC (mg kg^−1^soil)	818±4.57c	1038±9.32b	1270±20.12a
pH (H_2_O)	8.97±0.01a	8.72±0.01a	8.94±0.02a
Electrical conductivity(µS cm^−1^)	160±0a	170±0a	163±2a
Colonized root length (%)	29.72±4.53a	41.6±2.67a	53.79±9.61a

Degree of AM fungal colonization in roots of *R. communis*, *J. curcas* seedlings and native vegetation soil.

MBC: microbial biomass C; Means ± standard errors; Values in row followed by the same letter do not differ significantly (*P*<0.05) as determined by the LSD test.

The soil used to grow *J. curcas* showed a significantly-higher concentration of microbial biomass C than *R. communis* soil, but lower organic C.

The percentage of root colonisation for *J. curcas* and *R. communis* were average values 54 and 42%, respectively, without significant differences between the species. The roots present in the native vegetation soil showed less colonisation than those in soil planted with either crop species, but without significant differences (Table 1).

### PCR amplification of AMF SSU rRNA gene sequences from soil and diversity analyses

**Figure 1:**
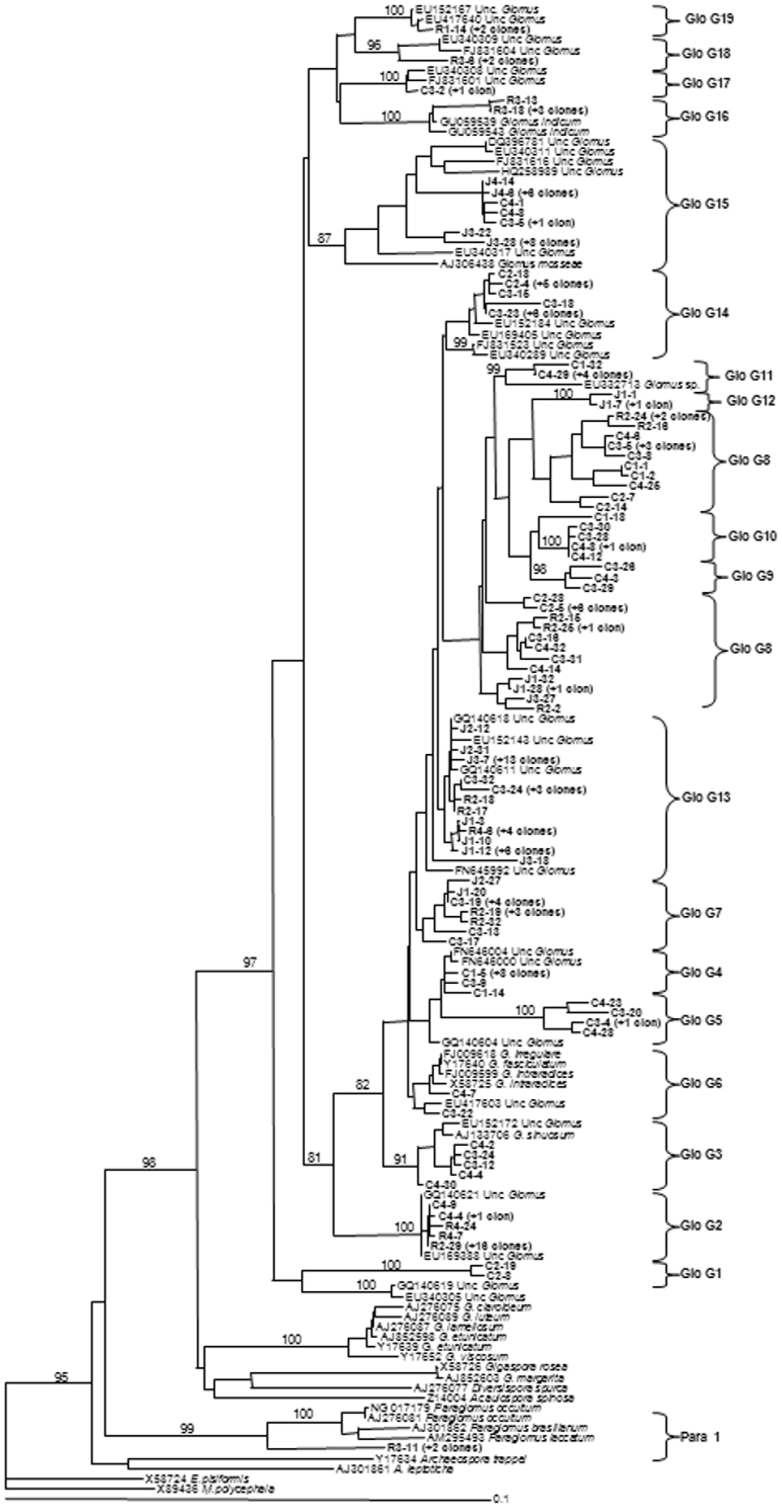
Neighbour-Joining (NJ) phylogenetic tree showing AM fungal sequences isolated from of native vegetation soil and *Ricinus communis* and *Jatropa curcas* soil and reference sequences from GeneBank. All bootstrap values >85% are shown (1000 replicates). Numbers above branches indicate the bootstrap values of the NJ analysis; numbers below branches indicate the bootstrap values of the maximum likelihood analysis. Sequences obtained in the present study are shown in bold type. They are labelled with the database accession number, the soil from the host plant from which they were obtained (C = native vegetation soil, R = *R. communis* soil and J = *J. curcas* soil) and the clone identity number. Group identifiers (for example Glo G1) are AM fungal sequences types found in our study. *Endogone pisiformis* and *Mortierella polycephala* were used as out-groups.

The PCR products obtained from the DNA in soil samples of native vegetation soil and soil planted with *J. curcas* or *R. communis* were used to construct SSU rDNA libraries. A total of 480 clones from 15 libraries were screened for the presence of the insert (32 clones/sample) and 320 clones resulted positive and were subsequently sequenced. BLAST analyses revealed that 206 sequences had a high similarity (96–100% identity) to AM fungal sequences (Table 2) and 114 to sequences of non-AMF origin, principally ascomycetes and basidiomycetes (data not shown); these were excluded from analyses. No chimeric sequences were detected. After phylogenetic analyses of the 206 sequences, 20 AM fungal sequence types or phylotypes could be distinguished on the basis of bootstrap values of least 85%. Of these, 19 belonged to the Glomeraceae and one to the Paraglomeraceae (Fig. 1). Since identical sequences were detected, the clones producing the same sequence for each host plant species soil sample and native vegetation soil were represented once in the alignment for clarity. A number of AMF sequence types were related to cultured AMF species. These were Para 1 for *Paraglomus laccatum-occultum-brasilianum*, Glo G3 for *Glomus sinuosum*, Glo G6 for *Glomus intraradices*-*G. fasciculatum-G. irregulare*, Glo G15 for *Glomus mosseae* and Glo G16 for *Glomus indicum*. Fifteen other AM fungal sequence types did not cluster with any sequence from AMF culture.

**Table 2:** Number of clones detected for each fungal type in the native vegetation soil and *Ricinus communis* and *Jatropa curcas* soil (*n* = 5).

				Total	
	Native vegetation soil	*R. communis s*oil	*J. curcas* soil	*n*	%
Para 1	-	3	-	3	1.46
Glo G1	2	-	-	2	0.97
Glo G2	3	19	-	22	10.68
Glo G3	5	-	-	5	2.43
Glo G4	11	-	-	11	5.34
Glo G5	5	-	-	5	2.43
Glo G6	2	-	-	2	0.97
Glo G7	7	5	2	14	6.80
Glo G8	23	8	4	35	16.99
Glo G9	3	-	-	3	1.46
Glo G10	6	-	-	6	2.91
Glo G11	6	-	-	6	2.91
Glo G12	-	-	3	3	1.46
Glo G13	5	7	26	38	18.45
Glo G14	16	-	-	16	7.77
Glo G15	4	-	18	22	10.68
Glo G16	-	5	-	5	2.43
Glo G17	2	-	-	2	0.97
Glo G18	-	3	-	3	1.46
Glo G19	-	3	-	3	1.46
**Total Clones**	**100**	**53**	**53**	**206**	**100**
**Total non-AMF Clones**	**27**	**31**	**56**	**114**	
**Total number of phylotypes**	**15**	**8**	**5**	**20**	**-**
**Shannon index**	**2.42**	**1.85**	**1.20**	**1.82**	**-**

The AMF diversity, expressed by the Shannon index, was higher in native vegetation soil (*H*′ = 2.42) than in *J. curcas* (*H*′ = 1.20) or *R. communis* (*H*′ = 1.85) soil. The native vegetation soil showed the highest number of AMF sequence types (15), only Glo G7, Glo G8 and Glo G13 being common to the soil of both crop species (Table 2). The *R. communis* soil had a richer AMF community than the *J. curcas* soil; of eight AMF sequence types found in the soil planted with *R. communis*, four (Para 1, Glo G16, Glo G18 and Glo G19) appeared exclusively here and four (Glo G2, Glo G7, Glo G8 and Glo G13) were found also in the native vegetation soil. The AMF sequence type Glo G12 was exclusive to the soil planted with *J. curcas*.

**Figure 2:**
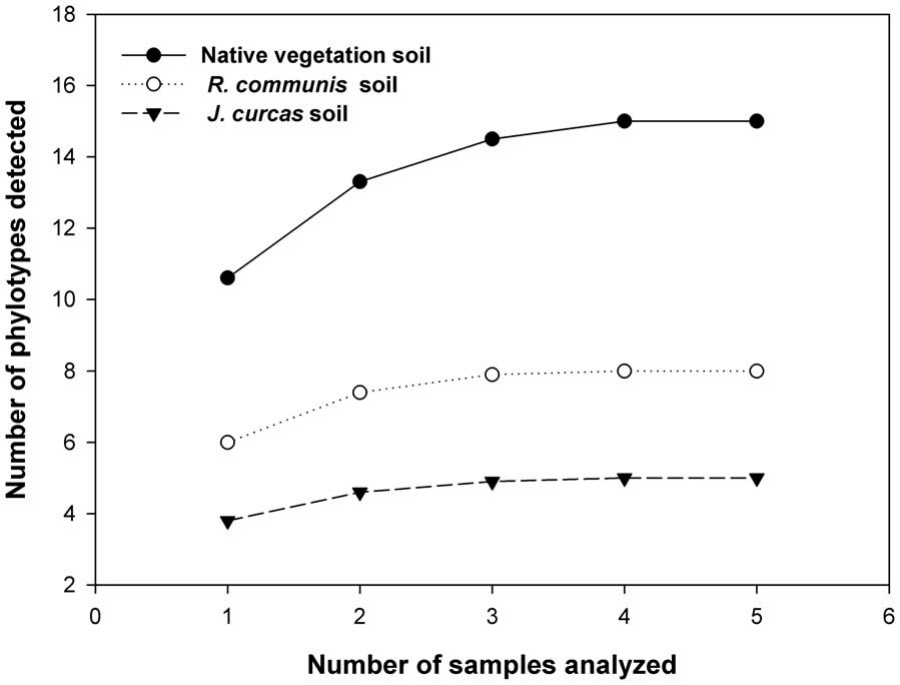
Sampling effort curves for native vegetation soil, *Ricinus communis* soil and *Jatropa curcas* soil. The sample order was randomized by 100 replications in EstimateS, version 8.0 [56].

The sampling effort curves (Fig. 2) showed that, in spite of the high number of non-AMF sequences found in this experiment, the number of samples analysed was sufficient to detect the majority of AMF sequence types present in the soils planted with *J. curcas* or *R. communis* and in the native vegetation soil samples, since the curves showed very-stable plateaus.

**Figure 3:**
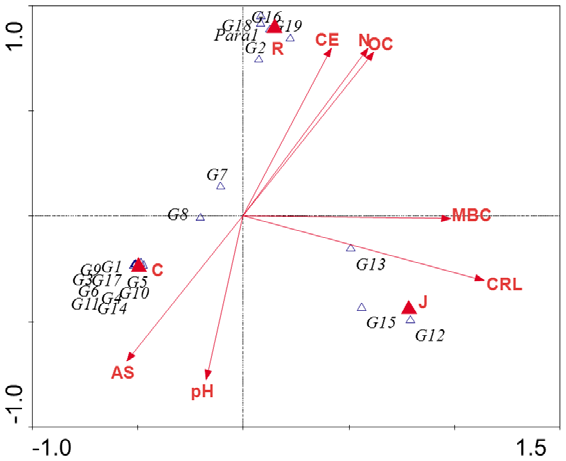
Canonical correspondence analysis (CCA) of the different AMF sequence types and soil properties detected in the three soils studied. The eigenvalues of the first and second axes in the two-dimensional ordination diagrams are as follows: CCA1: 0.59 and CCA2: 0.55. C: native vegetation soil, R: *Ricinus communis* soil, J: *Jatropa curcas* soil, MBC:.microbial biomass C, CRL: colonized root length, AS: aggregate stability, OC: organic C, N: total N, EC: electrical conductivity.

The distribution in the canonical correspondence analysis (CCA) diagram also shows that the AMF communities in the three soils were different: the communities from the soils of both crop species and the native vegetation soil grouped separately (Fig. 3).

### The relationship between the soil parameters measured, the root length colonised and the AMF diversity

The correlation analysis shows that only microbial biomass C was correlated negatively with AMF diversity, measured as the Shannon index (*P*<0.05) (Table 3). The rest of parameters did not show any significant correlation.

**Table 3:** Pearson's coefficients of correlation and significance level between the soil parameters measured, the root length colonised and the AMF diversity (n = 5).

Parameters	AMF diversity
Total N	−0.426 (0.720)
Organic C	−0.453 (0.701)
Aggregate stability	0.582 (0.605)
Microbial biomass C	−1.000 (0.014)
pH	0.072 (0.954)
Electrical conductivity	−0.256 (0.835)
Colonized root length	−0.973 (0.149)

## Discussion

This is the first study using molecular approaches of the diversity of the AMF in the root-associated soil after changing the land use to biofuel plant production. Our results clearly show a significant change in the composition of the soil AMF community when the two crop species were established. In the soils where *J. curcas* and *R. communis* grew, there were specific AMF communities. Several studies have shown host preferences of AMF in different habitats [16], [17], [18], [19], [20], [21], [22]. The AMF are totally dependent on the photosynthates provided by their host plant and therefore their survival is dependent on the host plant, so the host plant identity could have determined the AMF community composition detected in the soil of each crop species [23].

Although the mechanism involved in the differences in AMF community composition between the native vegetation soil and those of the two crop species is not actually known, it has been documented that various factors could be responsible for this selective interaction.

Several surveys [24], [25] have reported than cultivated soils have a lower AMF richness and diversity than non-cultivated soils (with native vegetation). In accordance with our study, the native vegetation soil had a higher Shannon index and number of AMF sequence types (H′ = 2.42) than *J. curcas* soil (H′ = 1.20) and *R. communis* soil (H′ = 1.85).

Other factors which could be influencing the AMF community composition are: competition for plant colonization among different species and/or strains, specificity in the interaction with the host plant and factors related with the soil conditions [26]. It is known that soil-related factors, such as pH, nutrient content, total soil C and N, humidity and temperature, may control and influence the diversity of both the plant and the fungi [27]. Also, Schreiner and Mihara [28] found that the diversity of AMF in grapevine roots was related to soil fertility. In our study, there was a significant improvement in the properties related to soil fertility (total N, aggregate stability and organic and microbial biomass C) when the two plant species were established (Table 1).

An increase in the soil fertility could have been due to the higher input of root exudates as a consequence of the establishment of crop plants. It has been documented that the soil organic C released by roots promotes a dense microbial community in the immediate environment of the root that can stimulate microbial activity and, in turn, reactivate microbial populations [29]. In fact, microbial biomass C has been used frequently as an indicator of soil microbial activity [30], [31] and is related closely to soil fertility [32]. Microbial biomass C was increased significantly in our study when the two crops were planted, the soil planted with *J. curcas* showing the highest value (Table 1). It is noteworthy that there was a negative correlation between microbial biomass C and AMF diversity measured as the Shannon index (*P*<0.05). This might be due to nutritional competition between non-AM fungi and AM fungi when the microbial populations are increased in their rhizospheres, since the DNA extracted from soil planted with the crops generated clones which provided non-AMF sequences, mainly from ascomycetes and basidiomycetes. It has been reported that these fungal groups colonise as many plant species and are as abundant as the AMF [33]. In this regard, the *J. curcas* soil had a higher number of non-AMF sequences and lower AMF diversity than the *R. communis* soil (Table 2).

With regard to AMF species distribution, several studies showed that *G. intraradices* is considered a cosmopolitan fungus that dominates the AMF community in different environments [18], [19], [24], [25], [26], [27], [34], [35], [36], [37]. In our study, surprisingly, Glo G6, that showed very-high similarity (99% identity) to *G. intraradices*, was not found in the soils planted with either crop species studied. It was found only in the native vegetation soil samples. We do not know of any study where the AMF diversity in *J. curcas* or *R. communis* has been investigated. In a study carried out with spores isolated from a soil in Thailand, whose aim was to check the mycorrhizal colonization of indigenous AMF of *J. curcas*, Charoenpakdee et al. [13] also did not find the presence of *G. intraradices*. We have no previous information about the AMF which colonise these crop species; *G. intraradices* may even be incompatible with them. Also, it might be, together with Glo G3 (98% identity with *Glomus sinuosum*), ruderal species [27].

It is interesting to note that only clones detected in *R. communis* soil showed high homology with *Glomus indicum*, a new arbuscular mycorrhizal fungus that was described recently by Blaszkowski et al. [38] in the rhizosphere of *Euphorbia heterophylla* growing naturally in coastal sands of Alappuzha (India) and in *Lactuca sativa* cultivated in Asmara (Africa). Another cultured AMF species found exclusively in *R. communis* soil was Para 1, that showed high similarity (97%) with *Paraglomus laccatum-occultum brasilianum* group. Glo G15, related to the *Glomus mosseae* group, was found in both native vegetation soil and soil planted with *J. curcas*, showing its greatest abundance in the latter case.

The rest of the AMF sequence types found in this study are related to database sequences from uncultured AMF detected in different host plant roots and ecosystems or are sequences which have been found for the first time in our survey.

In our study, the soil cultivated with *R. communis* had higher AMF diversity than the *J. curcas* soil. Since the diversity of AMF in the soil can affect the diversity and productivity of plants and, therefore, the stability, sustainability and functioning of the ecosystem [12], a major concern in biofuel plant production should be to ascertain whether the crop species affect the natural AMF diversity in the soil and thus the long-term sustainability of these agroecosystems.

In conclusion, our results show that some soil properties related to soil fertility showed higher values in *J. curcas* and *R. communis* soils, cultivated as biofuel crops in a tropical system. The diversity of the AMF community in the soils of both crops decreased with respect to the native vegetation soil and varied significantly depending on the crop species planted. Thus, *R. communis* soil showed higher AMF diversity than *J. curcas* soil, suggesting that this biofuel crop could be more suitable in long-term conservation and sustainable management of these tropical ecosytems; although more studies are needed in order to confirm this hypothesis.

## Materials and Methods

### Ethics Statement

No specific permits were required for the described field studies since these locations/activities belong to the “Instituto de Ecología y Sistemática”. The location is not privately-owned or protected in any way. Field studies did not involve endangered or protected species.

### Study Site

This research was conducted in the experimental area called “Paraguay” located in the province of Guantánamo (eastern Cuba) (coordinates 20°03′N, and 75°08′W). The climate is tropical, with an average annual rainfall of 800 mm and a mean annual temperature of 25°C. The soil type in the experimental area is an Inceptisol [39], and the natural vegetation in the zone was grassland dominated by *Jatropha gossypifolia* L., *Dichantium caricosum* L. A. Camus, *Cynodon nlenfuensis* Vanderyst and *Cynodon plectostachyus* (K. Schum) Pilg.

### Experimental design

Experimental plantations were conducted using a randomized factorial design with five replication blocks of 25×25 m. Three levels were established: two crop types: *Jatropha curcas* L. and *Ricinus communis* L., and non-planted areas (conserving the natural vegetation mentioned above) used as control. Initial seedlings spacing within plantations was 1 mand at least 40 seedlings per plant type and replication block were established. The soil was not tilled (seedlings were planted in individual holes). The native plants were removed when *J. curcas* and *R. communis* were established. The plants were manually pulled up trying not to leave roots in the soil. There was not management in the soil (fertilization, herbicide, ploughing), only weeding. The experiment started in November 2007. The weeding was performed whenever seedlings germinated appeared on the surface.

### Sampling

Two years and a half after plantation, in June 2010, soil samples for each treatment and replication block were taken (15 samples in total), each sample was a composite of 4 subsamples randomly taken at a depth of 10–30 cm and with a diameter of ca. 2.5 cm. The soil samples were taken from rhizospheres of biofuel plants, exactly below the plants (inside plants shade area). The soil samples were sieved through 2-mm pores to eliminate large particles and separate roots and then divided into two subsamples: one soil subsample was stored in plastic bags at −20°C for molecular analysis and another soil subsample was stored at 2°C to carry out the rest of analysis. At the same time, five root samples (aprox. 0.5 g fresh weight) per treatment and replication block (15 in total) were also harvested. In the case of native vegetation soil, the sampling was done in the grassland rhizosphere at random. These samples contained rhizospheric soil and roots.

### Mycorrhizal determinations

The percentage of mycorrhizal root colonization was estimated by visual observation of fungal colonization after clearing washed roots in 10% KOH and staining with 0.05% trypan blue in lactic acid (v/v), according to Phillips and Hayman [40]. The extent of mycorrhizal colonization was calculated according to the gridline intersect method [41]. At least 100 fields per sample were checked at 80× magnification.

#### Soil biological, chemical and physical analyses

Soil microbial biomass C was determined using a fumigation-extraction method [42]. Ten grams of soil at 60% of its field capacity are fumigated in a 125-ml Erlenmeyer flask with purified CHCl_3_ (Panreac) for 24 h placed in a glass desiccator. After removal of residual CHCl_3_, 40 ml of 0.5 M K_2_SO_4_ (Panreac) solution is added and the sample is shaken for 1 h before filtration of the mixture. The K_2_SO_4_-extracted C was determined with an automatic carbon analyser for liquid samples (Shimadzu TOC) and microbial biomass C is calculated as the difference between fumigated and non-fumigated samples.

Soil pH and electrical conductivity were measured in a 1∶5 (w/v) aqueous solution. Total N was determined with the Kjeldahl method, consisting of titration after distillation and sample digestion and the total organic C according to Yeomans and Bremner [43].

The percentage of stable aggregates was determined by the method described by Lax *et al.* [44]. A 4 g aliquot of sieved (0.2–4 mm) soil was placed on a small 0.250 mm sieve and wetted by spray. After 15 min the soil was subjected to an artificial rainfall of 150 ml with energy of 270 Jm^−2^. The remaining soil on the sieve was placed in a previously weighed capsule (T), dried at 105°C and weighed (P1). Then, the soil was soaked in distilled water and, after 2 h, passed through the same 0.250 mm sieve with the assistance of a small stick to break the remaining aggregates. The residue remaining on the sieve, which was made up of plant debris and sand particles, was dried at 105°C and weighed (P2). The percentage of stable aggregates with regard to the total aggregates was calculated by (P1-P2)×100/(4-P2+T).

### Soil DNA extraction and PCR

All PCR experiments were run using DNA preparations consisting of pooled soil extracts. DNA extractions from 15 soil samples were carried out.

For each of the 15 soil samples, genomic DNA was extracted from 0.5 g of soil fresh weight using a FastDNA™ Spin kit for soil according to the recommendations of the manufacturer (Q-BIOgene, Heidelberg, Germany). DNA extracts were stored at −20°C.

Several dilutions of extracted DNA (1/10, 1/50, 1/100) were prepared and 2 µl were used as template. Partial small subunit (SSU) ribosomal RNA gene fragments were amplified using nested PCR with the universal eukaryotic primers NS1 and NS4 [45]. PCR was carried out in a final volume of 25 µl using the “ready to go” PCR beads (Amersham Pharmacia Biotech), 0.2 µM dNTPs and 0.5 µM of each primer (PCR conditions: 94°C for 3 min, then 30 cycles at 94°C for 30 s, 40°C for 1 min, 72°C for 1 min, followed by a final extension period at 72°C for 10 min).

Two µl of several dilutions (1/10, 1/20, 1/50 and 1/100) from the first PCR were used as template DNA in a second PCR reaction performed using the specific primers AML1 and AML2 [46]. PCR reactions were carried out in a final volume of 25 µl using the “ready to go” PCR beads (Amersham Pharmacia Biotech), 0.2 µM dNTPs and 0.5 µM of each primer (PCR conditions: 94°C for 3 min, then 30 cycles of 1 min denaturation at 94°C, 1 min primer annealing at 50°C and 1 min extension at 72°C, followed by a final extension period of 10 min at 72°C). Positive and negative controls using PCR positive samples and sterile water respectively were also included in all amplifications. All the PCR reactions were run on a Perkin Elmer Cetus DNA Thermal Cycler. Reaction yields were estimated by using a 1.2% agarose gel containing ethidium bromide.

### Cloning and sequencing

The PCR products were purified using a Gel extraction Kit (Qiagen) cloned into pGEM-T Easy (Promega) and transformed into *Escherichia coli* (Xl1 blue). Thirty-two putative positive transformants were screened in each resulting SSU rRNA gene library, using 0.7 unit of RedTaq DNA polymerase (Sigma) and the supplied reaction buffer to a final volume of 25 µl and a re-amplification with AML1 and AML2 primers with the same cycling parameters described above. Product quality and size were checked in agarose gels as described above. All clones having inserts of the correct size in each library were sequenced.

Clones were grown in liquid culture and the plasmid extracted using the QIAprep Spin Miniprep Kit (Qiagen). The sequencing was done by Laboratory of Sistemas Genómicos (Valencia, Spain) using the universal primers SP6 and T7. Sequence editing was done using the program Sequencher version 4.1.4 (Gene Codes Corporation).

A search for chimeric sequences was performed using Chimera and Cross-Over Detection and Evaluation (Ccode) [47].

### Phylogenetical analysis

Sequence similarities were determined using the Basic Local Alignment Search Tool (BLASTn) sequence similarity search tool [48] provided by GenBank. Phylogenetic analysis was carried out on the sequences obtained in this study and those corresponding to the closest matches from GenBank. Sequences were aligned with other published glomeralean sequences using the program ClustalX [49] and the alignment was adjusted manually in GeneDoc [50]. Neighbour-joining (NJ) [51] and maximum likelihood (ML) phylogenetic analyses were performed with the programs PAUP4.08b [52] and RAxML v.7.0.4 [53], respectively. Distances for the NJ tree were computed using the default parameters. For the ML analysis, a GTR-GAMMA model of evolution was used. A total of 200 independent bootstrap analyses were performed to provide nodal support. The ML bootstrap values were calculated with 1000 replicates using the same substitution model. *Endogone pisiformis* Link and *Mortierella polycephala* Coem, were used as the out-groups.

Different AMF sequence types or phylotypes, were defined as groups of closely related sequences, usually with a high level of bootstrap support in the phylogenetic analyses (higher than 85%) and sequence similarity ≥97%. The pairwise analysis within clusters was carried out using MEGA software version 4 [54].

Seventy-nine representative sequences of the clones generated in this study have been deposited at the National Centre for Biotechnology Information (NCBI) GenBank (http://www.ncbi.nlm.nih.gov) under the accession numbers FR821524–FR821602.

### Diversity of AM fungal community

The Shannon (H′) index was calculated as an additional measure of diversity, as it combines two components of diversity, i.e., species richness and evenness. It is calculated from the equation H′ = −∑*p_i_*(ln *p_i_*), where *pi* is the proportion of individuals found in the *_i_*th species (in a sample, the true value of *p_i_* is unknown but is estimated as *n_i_*/*N*, [here and throughout, *n_i_* is the number of individuals in the *_i_*th species]).

### Statistical analysis

Aggregate stability and percentage colonization were arcsine-transformed, and the other parameters were log-transformed to compensate for variance heterogeneity before analysis of variance. The data (colonization and soil properties) were subjected to analysis of variance, and comparisons among means were made using a Least Significant Difference (LSD) test calculated at *p*<0.05. Correlation analysis between all the soil parameters measured, colonized root length and the AMF diversity was carried out using Pearson's rank correlation coefficients. Statistical procedures were carried out with the software package IBM SPSS Statistic 19.0 for Windows.

In order to investigate the influence of change of land use on the AM fungal community composition and soil properties, ordination analyses were conducted in CANOCO for Windows v. 4.5 [55] using the relative abundance data for each AMF sequence type. Initial detrended correspondence analysis suggested a unimodal character of the data response to the sample origin (the lengths of gradients were >4); therefore, the canonical-correspondence analysis (CCA) was used. Monte Carlo permutation were conducted using 499 random permutations.

The presence or absence of AMF phylotypes in each soil sample was used to construct the sampling effort curves (with 95% confidence intervals) using the software EstimateS 8.00 [56]. The sample order was randomized by 100 replications.
